# The Spatial Organization of the Intranuclear Structures of Human Brain Dopaminergic Neurons

**Published:** 2017

**Authors:** D. E. Korzhevskii, V. V. Gusel’nikova, O. V. Kirik, E. G. Sukhorukova, I. P. Grigorev

**Affiliations:** Federal State Budgetary Research Institution «Institute of Experimental Medicine», akad. Pavlov Str. 12, St. Petersburg, 197376, Russia

**Keywords:** brain, dopaminergic neurons, human, Marinesco body, nucleolus, nucleophosmin, substantia nigra, ubiquitin

## Abstract

We studied the intranuclear localization of protein nucleophosmin (B23) and ubiquitin in the dopaminergic neurons of human substantia nigra (n = 6, age of 25–87 years) using immunohistochemistry and confocal laser microscopy. Intranuclear ubiquitin-immunopositive bodies that morphologically correspond to Marinesco bodies were found to be present in substantia nigra dopaminergic (tyrosine hydroxylase-immunopositive) neurons but absent in non-dopaminergic neurons. The number of bodies varied from 0 to 6 per cell nucleus. Nucleophosmin (B23) was found in the neuronal nucleolus, with the nucleolus size being constant in the nigral neurons of each individual brain. All the observed neurons had only one large nucleolus with intense nucleophosmin immunoreactivity and a lightly stained region (1–2 μm in diameter) that apparently represents the giant fibrillar center (GFC). An intensely immunostained nucleophosmin-containing granule was often observed at the GFC periphery. Double labeling demonstrated that nucleophosmin-immunoreactive nucleolus and ubiquitin-immunoreactive Marinesco bodies can occur both closely to and remotely from each other. Three-dimensional reconstruction indicates that rounded Marinesco bodies are polymorphic and often have a complex shape, with some flattening and concavities, which may be associated with contact not only with the nucleolus, but also, presumably, with other intranuclear structures free of ubiquitin or nucleophosmin. Ubiquitin-immunoreactive structures with a relatively small size (up to 1 μm in length) and various clastosome-like shapes (Lafarga et al., 2002) often occur near Marinesco bodies. There were no cases of detection of ubiquitin in the nucleoli of dopaminergic neurons and nucleophosmin/B23 in typical Marinesco bodies. The obtained information may be helpful in unraveling the molecular mechanisms of the selective vulnerability of substantia nigra dopaminergic neurons to damaging factors.

## INTRODUCTION

The eukaryotic cell nucleus is characterized by a complex internal structural and functional compartmentalization that enhances the effectiveness of intracellular processes by concentrating specific factors in certain nuclear regions. The most important nuclear compartments include nucleoli, nuclear speckles, Cajal bodies, PML bodies, etc. [1]. In this case, some intranuclear structures (e.g., nucleoli) are present in most eukaryotic cells, while others are characteristic of a specific cell type. An example of such specific intranuclear structures is the Marinesco bodies that normally occur exclusively in the neurons of substantia nigra and locus coeruleus in the brain of humans and primates [2, 3]. Despite the fact that both nucleoli and Marinesco bodies are individually well characterized structures, their shape and spatial relationship in the nuclei of nerve cells have not been explored in detail. This problem may be solved using confocal microscopy involving the use of primary antibodies to known marker proteins of various intranuclear structures. For the nucleolus, this marker is nucleophosmin (NPM, B23), a polyfunctional protein that is involved in ribosome biogenesis, centrosome duplication, and the regulation of proliferation and apoptosis [4-6]. Interestingly, B23 is expressed at a high level not only in actively proliferating cells, but also in postmitotic neurons, but its role in these neurons is actually unknown [6]. In the nucleolus of nerve cells, B23 is supposed to act as a cellular stress sensor initiating the mechanisms that promote the maintenance of neuronal viability (e.g., by stabilizing the transcription factor p53) [7]. In addition to that, a normal level of B23 in neurons is probably a condition for maintaining blockade of the cell cycle, while excessive expression of the protein may cause the cell to return to the cell cycle and initiate neuronal death, which is observed in neurodegenerative diseases such as Alzheimer’s disease, Parkinson’s disease, and amyotrophic lateral sclerosis [8-10]. In this regard, an immunocytochemical study of the B23 protein distribution in substantia nigra dopaminergic neurons of the human brain is particularly important, because mass death of dopaminergic neurons is a characteristic feature of Parkinson’s disease and an indispensable indicator of the adequacy of experimental models of this disease [11, 12].

In contrast to B23, whose distribution features in dopaminergic neurons have not been explored, ubiquitin, a component of the proteasome system responsible for degrading damaged proteins, has been repeatedly studied when analyzing the functional state of substantia nigra neurons in normal and pathological conditions [13, 14]. Currently, ubiquitin is considered as a specific marker of Marinesco bodies; the functional significance of the latter still remains unknown [3]. Studies of intranuclear ubiquitin-immunopositive bodies of substantia nigra neurons of the human brain using light microscopy and immunocytochemistry have demonstrated that up to 20% of substantia nigra neurons can contain ubiquitin-immunoreactive bodies whose morphological characteristics are similar to those of Marinesco bodies [15]. In the same studies, the nuclei of substantia nigra neurons were found to comprise structures that differ from Marinesco bodies in a number of characteristics but contain ubiquitin in a detectable amount [15]. In recent years, reports have appeared indicating that ubiquitin (along with ubiquitin-like proteins) plays an important role not only in intracellular protein degradation processes, but also in ribosome biogenesis [16]. This suggests the presence of this protein in the nucleolus, but the results of immunocytochemical studies, which would confirm this suggestion, are absent.

Therefore, investigation of the shape and spatial relationship of B23- and ubiquitin-immunopositive structures in dopaminergic neurons of the human brain is a topical issue of modern neuroscience and is of great interest in basic neurology. Therefore, the search for approaches to solving these issues was the purpose of the present work.

## EXPERIMENTAL

We used fragments of the human midbrain (*n *= 6, males and females aged 25 to 87 years who died of reasons not related to brain diseases and damage). The material was received from the archive of the Department of General and Private Morphology of the Institute of Experimental Medicine. The research program was approved by the Local Ethical Committee of the Institute of Experimental Medicine.

The material was fixed in zinc-ethanol-formaldehyde [17] and immersed into paraffin. Paraffin blocks were used to prepare slices with a thickness of 5, 7, and 10 μm, which were pasted onto slides with an adhesive coating (Histobond, Polysine, SuperFrost Gold, Germany). A fraction of the samples were stained using a classical neurohistological technique – Nissl staining with toluidine blue. Before starting immunocytochemical reactions, the material was verified for suitability to analysis (a neurodegenerative process and post-mortem autolysis were excluded). To improve the immunoreactivity of detected antigens, they were thermally retrieved in modified citrate buffer, pH 6.1 (S1700, Dako, Denmark). Control immunohistochemical reactions were carried out with allowance for the recommendations of the reagent’s manufacturer.

When performing immunohistochemical reactions for transmitted light microscopy, we used the following primary antibodies: rabbit polyclonal anti-ubiquitin antibodies (Dako) at a 1 : 400 dilution; mouse monoclonal anti-B23 (nucleophosmin) antibodies, clone FC82291 (Sigma-Aldrich, USA), at a 1 : 200 dilution; mouse monoclonal anti-tyrosine hydroxylase antibodies (clone 1B5) at a 1 : 50 dilution (Leica-Novocastra, UK). A MACH_2_-Universal HRP-Polymer reagent (Biocare Medical, USA) was used to identify the rabbit and mouse primary antibodies associated with the studied markers. The peroxidase label was detected using diaminobenzidine chromogen (DAB+; Dako). After performing immunocytochemical reactions, a fraction of the sections were stained with a 0.5% aqueous cresyl violet solution (Dr. Grubler, Germany) and a 0.1% aqueous astra blue solution (Merck, Germany).

When performing individual and combined immunohistochemical reactions for confocal laser microscopy, we used the same primary antibodies as for the immunoperoxidase reaction. In a double reaction, we used two antibody combinations antibodies to tyrosine hydroxylase/ubiquitin and antibodies to B23/ubiquitin. After thermal retrieval of antigens, they were incubated with primary antibodies at 27 °C for 65 h. A biotin-labeled monovalent Fab fragment of donkey immunoglobulin (Jackson ImmunoResearch, USA) was used as a secondary antibody to detect primary mouse antibodies. After treatment with secondary antibodies, the samples were incubated in a solution of streptavidin conjugated with a Cy2 fluorochrome (Jackson ImmunoResearch). Primary rabbit antibodies were detected with pig anti-rabbit immunoglobulin antibodies conjugated with tetramethylrhodamine isothiocyanate (TRITC) produced by Dako. After performing a single reaction for the B23 protein, a fraction of the samples were stained with a 7-AAD nuclear dye (Invitrogen, USA). Confocal microscopy was performed using a LSM 710 microscope (Carl Zeiss, Germany).

After performing the immunocytochemical reaction for B23, we determined the nucleolus size in substantia nigra neurons. The nucleolus diameter was measured using the LAS EZ software (Leica, Germany). We analyzed nucleoli only in neurons where neuromelanin granules were clearly visible in the cytoplasm. The measurements were performed independently by two investigators (O.V. Kirik and V.V. Gusel’nikova) on two different Leica DM750 microscopes (Leica) equipped with ICC50 and ICC50HD cameras (Leica), after additional calibration of the system using an object micrometer. Quantitative data were processed in Excel software (Microsoft, USA) and represented as the mean (X) and standard deviation (σ). The coefficient of variation (V) was calculated to evaluate population homogeneity.

## RESULTS AND DISCUSSION

**Fig. 1:**
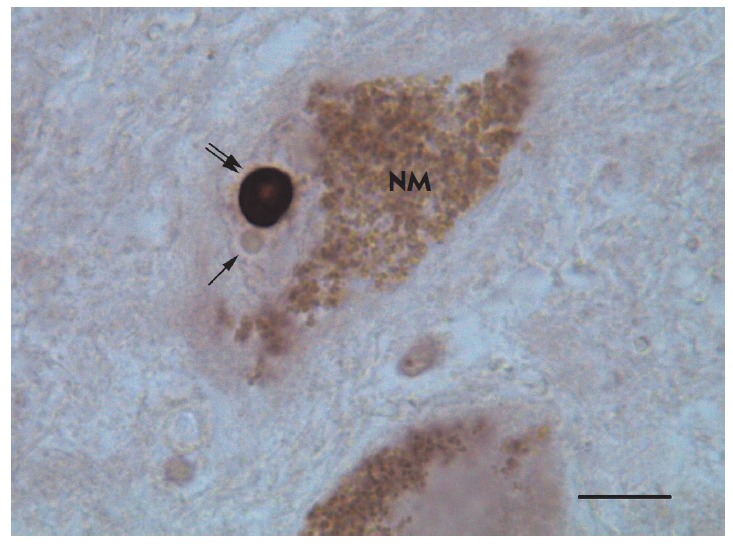
A dopaminergic neuron with a B23-immunopositive nucleolus in human substantia nigra. NM – neuromelanin granules in the neuronal cytoplasm; the single arrow indicates an unstained Marinesco body; the double arrow indicates the immunopositive nucleolus. Protein B23 immunocytochemistry without counterstaining. Plan objective lens 100×/1.25 Oil (oil immersion). Eyepiece HC Plan s 10×/18. Scale bar=10 μm.

**Fig. 2:**
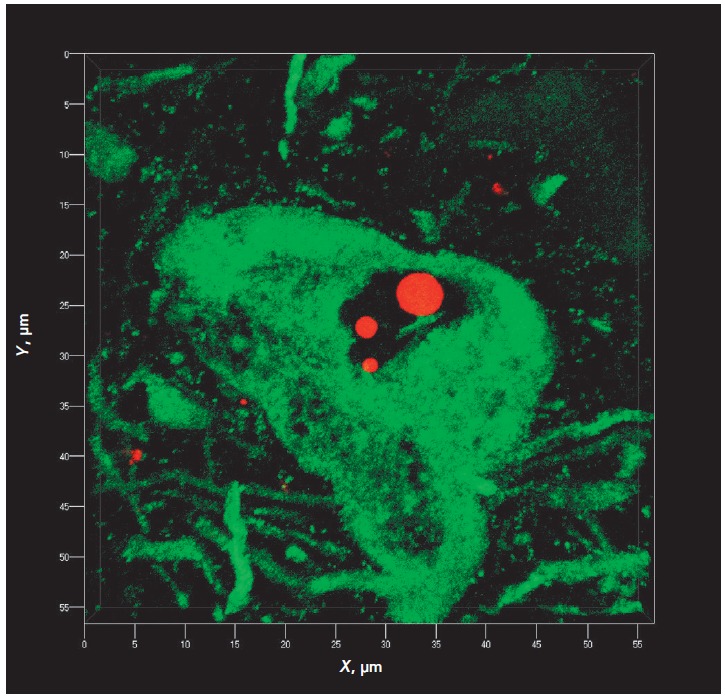
Ubiquitin-immunopositive structures in human substantia nigra. An immunopositive response to ubiquitin (red) is provided by three rounded Marinesco bodies in the neuronal nucleus and some granules in the substantia nigra neuropil. Double immunocytochemistry for tyrosine hydroxylase is visualized with fluorochrome Cy2 (green), and that for ubiquitin is visualized with fluorochrome TRITC (red). Confocal laser microscopy. 3D reconstruction in shadow projection is carried out using a ZEN 2011 software module (Zeiss, Germany). Z-stack covers a depth of 5.6 μm and 29 optical sections. Objective lens 100×/1.40 Oil DIC M27 (oil immersion).

In all cases, neuronal nucleoli (both in Nissl staining with toluidine blue and in the B23 protein test) and ubiquitin-immunopositive bodies were found in the samples (*Fig. 1*). A double immunofluorescence reaction for tyrosine hydroxylase and ubiquitin (*Fig. 2*) showed that intranuclear ubiquitin-immunopositive bodies were actually present in substantia nigra dopaminergic neurons and were absent in the neurons not responding to a marker enzyme of catecholamine synthesis, tyrosine hydroxylase.

Visualization of nucleoli using the reaction for nucleophosmin (B23) revealed the heterogeneity of their structure, with smooth contours and a rounded shape being constant. The presence of additional nucleoli was found not to be typical of substantia nigra neurons. All observed neurons contained only one large nucleolus brightly stained in the reaction for nucleophosmin; the nucleolus often contained a slightly stained region. A similar structure, which had been previously called nucleolus vacuole, was regularly found in the nucleoli of large neurons [18]. Later, the structure was found to be a giant fibrillar center (GFC) containing, predominantly, the UBF factor [19]. Interestingly, the nucleoli of neurons from different subjects from the study sample were characterized by a degree of individuality in size and a fairly low variability in size (*Table*).

**Fig. 3:**
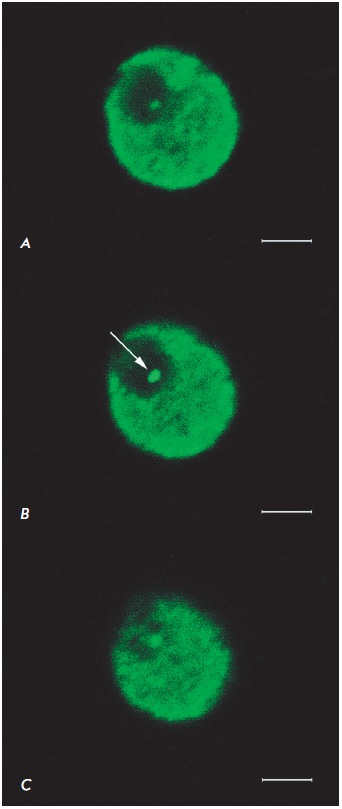
The nucleolus of a human substantia nigra dopaminergic neuron. Consecutive optical sections with a 0.4 μm interval. The arrow indicates an immunopositive granule near a giant fibrillar center. Protein B23 immunocytochemistry is visualized with fluorochrome Cy2 (green). Confocal laser microscopy. Plan-Apochromat objective lens 100×/1.40 Oil DIC M27 (oil immersion). Scale bar=2 μm.

The investigation of the nucleolus using confocal microscopy confirmed the accuracy of the measuremets performed on the immunoperoxidase samples. In this case, a potential false increase in the size of the studied structure due to chromogen diffusion was excluded. The giant fibrillar center that was usually located at the nucleolus periphery was found to reach 1–2 μm in diameter. The GFC area was characterized by weak fluorescence in the reaction for B23, which indicates a reduced concentration (but not an absence) of the protein in this nucleolar compartment. The peripheral GFC portion often contained a brightly fluorescing granule concentrating the B23 protein (*Fig. 3*).

**Table:** Measurements of the nucleoli of neuromelanin-containing substantia nigra neurons

Case	Mean diameter (X), μm	Standard deviation, σ	Variation coefficient (V), %
Male, 25 y.o.	5.1	0.6	11.3
Male, 51 y.o.	4.2	0.4	8.6
Male, 61 y.o.	5.9	0.5	8.9
Female, 62 y.o.	6.1	0.3	4.9
Female, 78 y.o.	5.6	0.3	5.6
Male, 87 y.o.	5.6	0.4	6.8

**Fig. 4:**
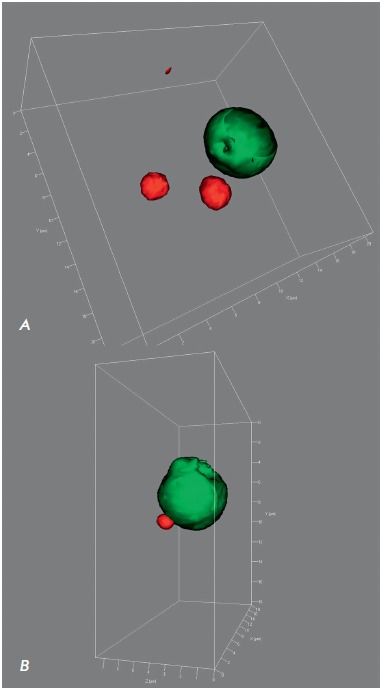
3D reconstruction of intranuclear structures of human substantia nigra dopaminergic neurons. Double immunocytochemistry for the B23 protein is visualized with fluorochrome Cy2 (green, stained nucleolus), and that for ubiquitin is visualized with fluorochrome TRITC (red). Confocal laser microscopy. 3D surface reconstruction is carried out using a ZEN 2011 software module (Zeiss, Germany). Z-stack covers a depth of 9.8 μm (A) and 7.6 μm (B); the number of optical sections is 50 (A) and 39 (B). Plan-Apochromat objective lens 100×/1.40 Oil DIC M27 (oil immersion).

The double B23 protein and ubiquitin reaction clearly visualized the nucleolus and Marinesco bodies. A high fluorescence intensity during the detection of both markers enabled adequate three-dimensional reconstruction of the studied structures, both in the translucent object mode and in the object surface contour mode (*Fig. 4*). Spatial reconstruction of nucleoli and Marinesco bodies revealed that not all observed objects had a regular spherical shape. For example, ball or ellipsoid shapes were typical of the nucleoli, but single nucleoli had pear and dumbbell shapes.

**Fig. 5:**
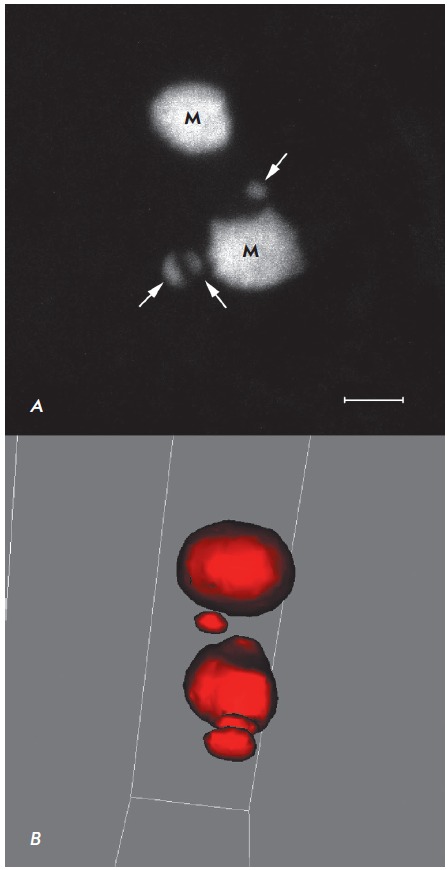
Marinesco bodies in the nucleus of a dopaminergic neuron of human substantia nigra. M – Marinesco bodies; arrows – ubiquitin-immunopositive structures that are not typical Marinesco bodies. Confocal laser microscopy. A – the image is a superposition of 30 optical sections made with a 0.2 μm interval; B – 3D surface reconstruction rotated by 90° is carried out using a ZEN 2011 software module (Zeiss, Germany). Z-stack covers a depth of 5.8 μm; the number of optical sections is 30. Plan-Apochromat objective lens 100×/1.40 Oil DIC M27 (oil immersion). Scale bar = 2 μm.

Marinesco bodies were characterized by high polymorphism, but they always had clear contours (*Fig. 5*). These bodies were present in neuronal nuclei in different amounts (up to six within the nucleus of one cell) and occupied a different position relative to the nucleolus. For example, the body could be closely associated with the nucleolus and be immediately adjacent to its surface, but in most cases, the body was located at a short distance from the nucleolus or was remote from it. If one nucleus contained several Marinesco bodies, the bodies could be both remote from each other and grouped, sometimes with direct contact of the boundaries. The localization of several bodies relative to the nucleolus was also different. We observed cases where a fraction of the bodies were immediately adjacent to the nucleolus boundary, while other bodies were remote from it. Sometimes, bodies surrounded the nucleolus from different directions. In some cases, all bodies occurred at a considerable distance from the nucleolus. The use of 3D reconstruction enabled a visualization of all shape details of the detected Marinesco bodies (*Fig. 5*). Our findings indicate that most of the identified bodies had a regular rounded, and less often oval, shape. However, in some cases, flattened or concave regions were present on the surface of detected bodies. The formation of this complex surface pattern of the Marinesco body may be caused by the presence of another structure directly adjacent to the surface of this body. Indirectly, this is confirmed by our earlier data indicating that flattened or concave regions of ubiquitin-immunopositive bodies can form on the body surface at the contacts of the bodies with the nucleolus [15]. Furthermore, in several cases, there were ubiquitin-immunopositive bodies adjacent to the surface of a structure that we defined as an additional nucleolus; flattening or concavity was also formed on the surface of these bodies [15]. However, the double ubiquitin and B23 reaction demonstrated that, in some cases, Marinesco bodies are remote from the B23-positive nucleolus but still have a complex surface shape. This may be an indication that the nuclei of substantia nigra neurons may contain other structures that interact with Marinesco bodies. On the other hand, the irregular contour of these bodies in the absence of a bounding membrane may reflect the dynamics of macromolecules and be a result of an escape of molecules from the peripheral parts of Marinesco bodies.

Another important result obtained by confocal microscopy with layer scanning and 3D reconstruction was a detailed description of the morphology of specific ubiquitin-positive structures that, for a number of reasons, cannot be considered as Marinesco bodies but are clearly identified by an appropriate reaction for ubiquitin (*Fig. 5*). These structures are of a relatively small size (up to 1 μm in length) and of various shapes (round, oval, rod-shaped, etc.). Like Marinesco bodies, these structures are characterized by the variability of their distribution within the nucleus, with the structures being often located near typical Marinesco bodies and sometimes adjoining them. It is worth noting that when these ubiquitin-positive structures directly adjoin typical Marinesco bodies, the surface of the latter contains flattened or concave areas facing this structure, which indirectly confirms the suggestion that the complex surface of Marinesco bodies forms non-randomly. The nature of the identified ubiquitin-immunopositive structures that are not Marinesco bodies remains unknown. In this regard, of great importance are the data presented by M. Lafarga et al., who found (using confocal and electron microscopy) special structures, calledclastosomes, in the nuclei of several cell types [20]. According to [20], these intranuclear structures contain ubiquitin at a high concentration and are the site of destruction of various proteins. In this case, the presence of clastosomes in cell nuclei is determined by the intensity of the proteasome degradation processes in the cell: the more intense the processes, the more pronounced the clastosomes [20]. This circumstance could explain the presence of ubiquitin-positive structures only in single substantia nigra neurons, along with their absence in most cells, by the different functional states of the analyzed neurons.

**Fig. 6:**
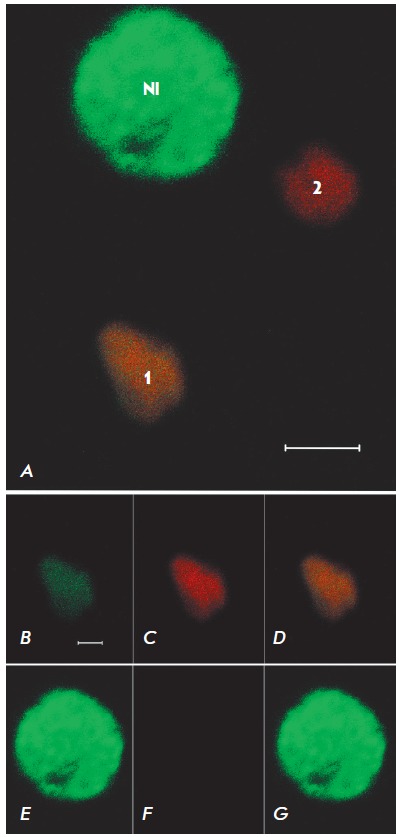
Colocalization of the B23 protein and ubiquitin in the nucleus of a human substantia nigra dopaminergic neuron. A – a general view showing the immunopositive reaction of nuclear structures; B – colocalization of the B23 protein and ubiquitin in a typical Marinesco body (structure 1). Nl – a B23-immunopositive neuronal nucleus (without colocalization with ubiquitin), structure 2 – a typical Marinesco body (without colocalization of the studied proteins). B, E – a green channel (protein B23). C, F – a red channel (ubiquitin). D, G – an aligned image. Double immunocytochemistry for the B23 protein is visualized with fluorochrome Cy2 (green), and that for ubiquitin is visualized with fluorochrome TRITC (red). Confocal laser microscopy. Plan-Apochromat objective lens 100×/1.40 Oil DIC M27 (oil immersion). Scale bars = 2 μm (A) and 1 μm (B–G).

An investigation of the colocalization of two proteins (ubiquitin and B23) in the nucleoli of dopaminergic neurons and Marinesco bodies demonstrated the presence of the B23 protein and the absence of ubiquitin in the nucleolus. The B23 protein never colocalizes with ubiquitin in the nucleolus. Even when ubiquitin-immunopositive bodies are in direct contact with the nucleolus (*Fig. 4B*), the area of apparent colocalization does not exceed the resolution of the used equipment (0.2 μm). In contrast to the nucleolus, the colocalization of ubiquitin and the B23 protein in Marinesco bodies is atypical but possible (*Fig. 6*). In this case, the fluorescence of B23 is much weaker than that in the area of the intensely stained nucleolar regions and is comparable to the fluorescence of the GFC region. The identification of bodies where the B23 protein is present and colocalized with ubiquitin in the neuronal nuclei raises a question as to the nature of these structures. There is evidence that ubiquitin (along with ubiquitin-like proteins) plays an important role not only in intracellular protein degradation processes, but also in ribosome biogenesis [16], which suggests the presence of this protein in the nucleolus. However, as seen from *Figure 6*, the identified B23/ubiquitin-immunopositive structure is characterized by an irregular shape, as well as by the lack of internal structuredness and a GFC region; therefore, it cannot be defined as the nucleolus, especially given the above data on the absence of additional nucleoli in substantia nigra dopaminergic neurons. The peculiarities of the shape and size of the identified bodies, as well as the high concentration of ubiquitin in them, rather indicate that these intranuclear structures are a specific type of Marinesco bodies that contain the B23 protein. However, we cannot exclude the fact that the detected B23/ubiquitin-immunopositive structures could be independent intranuclear inclusions that are not related to Marinesco bodies or clastosomes.

## CONCLUSION

Our findings indicate that the nuclei of dopaminergic neurons of the human substantia nigra comprise several types of structures that contain the studied proteins and have various shapes. The clastosome-like structures are characterized by relatively small sizes (up to 2 μm in diameter), a regular shape, and location near the nucleolus. At different distances from the nucleolus, there are larger polymorphic Marinesco bodies (usually 2–4 μm in diameter) that include atypical structures containing both ubiquitin and the B23 protein. The largest and most constant structure of the nucleus is the nucleolus. We have demonstrated the monomorphism and stability of the nucleolus size in human substantia nigra neurons. The nucleolus of dopaminergic neurons was found by us to be characterized by the presence of a giant fibrillar center (GFC) that had been previously studied in detail only in the neurons of laboratory animals. We have demonstrated that human GFC, unlike rat GFC, includes a non-constant microstructure containing the B23 protein.

All of these facts provide new information on the dopaminergic neurons of the human brain. Further research in this area to investigate the spatial relationship of the nucleolus and Marinesco bodies with other intranuclear structures (Cajal bodies, PML bodies, nuclear speckles), as well as the dynamics of these structures in neurodegeneration, will elucidate how the intranuclear structures are involved in the regulation of the functional state of catecholaminergic neurons. Studying the distribution of the proteins comprised in these structures in norm and pathology may facilitate the identification of new molecular markers of the neurodegeneration process. The analysis of the intranuclear structures of neurons resistant to damaging factors will demonstrate the presence (or absence) of a relationship between the features of intranuclear inclusions and the selective sensitivity of substantia nigra dopaminergic neurons to damage.
